# *Treponema pallidum* infection among women with macerated stillbirth in Mwanza Tanzania: an underestimated tragedy in Tanzania

**DOI:** 10.4314/ahs.v22i3.9

**Published:** 2022-09

**Authors:** Rose A Malisa, Edgard Ndaboine, Elieza Chibwe, Fridolin Mujuni, Helmut Nyawale, Majigo Mtebe, Stephen E Mshana, Mariam M Mirambo

**Affiliations:** 1 Shinyanga Referral Regional hospital, P.O.Box 17, Shinyanga, Tanzania; 2 Department of obstetrics and gynecology, Catholic university of health and Allied Sciences, P.O.Box 1464, Mwanza, Tanzania; 3 Department of Microbiology and Immunology, Catholic university of health and Allied Sciences, P.O.Box 1464, Mwanza, Tanzania; 4 Department of Microbiology and Immunology, Muhimbili University of Health and Allied Sciences, P.O.Box 65001, Dar es Salaam, Tanzania

**Keywords:** *Treponema pallidum*, macerated stillbirth, Mwanza, Tanzania

## Abstract

**Background:**

*Treponema pallidum* is one of the commonest cause of stillbirths world-wide. This study investigated the magnitude of *T. pallidum* among women with macerated stillbirth in Mwanza, Tanzania.

**Methods:**

A cross-sectional study involving 301 women with macerated stillbirths attending selected health facilities in the region of Mwanza was conducted between October-2017 and March-2018. Detection of *T. pallidum* was done using venereal diseases research laboratory (VDRL) and *T. pallidum* hemagglutination test (TPHA). Data were analyzed by the STATA version 13.

**Results:**

The median age of the enrolled women was 27 (IQR: 22 - 34) years. Eighteen (6.0%, 95% CI: 3.0–8.0) of women were *T. pallidum* seropositive. Seropositivity of *T. pallidum* was significantly higher among women residing in rural areas than urban areas (p=0.010), and among HIV seropositive than HIV seronegative women (p=0.036). By multivariable regression analysis, the odds of being *T. pallidum* seropositive were significantly high among women with positive HIV serostatus (OR: 3.9, 95% CI: 1.2–14.1, p=0.036) and those residing in rural areas (OR: 5.6, 95% CI: 1.5–20.3, p=0.010).

**Conclusion:**

Seropositivity of *T. pallidum* is higher among women with macerated stillbirth than in normal pregnant women as previously reported which calls for the need to improve screening services in rural areas of Tanzania.

## Background

Stillbirth (SB) is one of the most common adverse pregnancy outcome, occurring in an estimated 2 million cases each year worldwide with more than 98% of cases occurring in low and middle income countries (LMICs) 1–4. In LMICs, stillbirths rates ranges from 20 to 40 per 1000 births, with rates as high as 100/1000 in some areas, compared to 3 to 5 per 1000 births in high income countries (HICs) 5–8. In many HICs, there has been a significant reduction in stillbirths, over the past decades mostly due to improvement in medical care while rates in LMICs have remained high 9.

Tanzania is among the top 10 countries with high number of stillbirths globally with approximately 47,000 stillborn babies each year 10. Almost half of these stillbirths (22,000) occurs during delivery, indicating a critical need for better quality of care around birth. Like other adverse pregnancy outcomes that have been reported in Tanzania 11–13, stillbirth is a major public concern with multifactorial causes including infections. In this setting, much has been done to reduce fresh stillbirth (FSB) rate from 19 per 1000 to 14.5 per 1000^14^, however, little has been done on macerated stillbirth (MSB) which is known to be associated with bacterial infections 3. In HICs maternal infections accounts between 10 and 25 % of stillbirth cases while in LMICs it accounts for more than 50% 3, 7, 15. Syphilis, a disease caused by *Treponema palladium* has been associated with SB and is prevalent in LMICs including Tanzania 16, 17. In Tanzania the prevalence of maternal syphilis has been found to range from 2.5% to 17% among pregnant women 18–20.

Previous studies have reported syphilis seroconversion rate of 2.7% and 3.1% in Mwanza and Arusha, respectively during the course of pregnancy 21. If not screened, syphilis can cause stillbirth in 51% of cases and if not treated it may account for up to 94% of stillbirth cases 18. In a view of that, this study was conducted to provide baseline data and create more evidence-based information regarding the magnitude of syphilis in pregnant women presenting with MSB.

## Methods

### Study design, duration and study area

Cross-sectional hospital-based study was conducted in selected health facilities in urban and rural areas in the region of Mwanza between October 2017 and March 2018. The study enrolled patients from the Bugando Medical Centre (BMC), Sekou Toure Regional Referral hospital, Nyamagana District Hospital, Sengerema Designated District Hospital, Bukumbi Mission hospital, Misungwi district hospital, Ngudu district hospital, Sumve Mission hospital and Magu district hospital (Table 1).

**Table 1:** Summary of hospitals 1 involved in this study

SNO	Hospital	Status	Location	Deliveries per month	Stillbirth per month	Recruited
1	Bugando Medical Centre	Private	Nyamagana	740	10	42
2	Sekou Toure	Public	Nyamagana	700	20	99
3	Nyamagana District hospital	Public	Nyamagana	449	15	9
4	Magu district hospital	Public	Magu	230	9	42
5	Sumve hospital	Private	Misungwi	130	3	9
6	Sengerema hospital	Private	Sengerema	746	27	63
7	Misungwi Hospital	Public	Misungwi	200	4	12
8	Bukumbi Hospital	Private	Misungwi	82	2	3
9	Ngudu District Hospital	Public	Kwimba	245	7	21

### Study population and selection criteria

During the study period 21,151 pregnant women from 28 weeks gave birth in the selected health facilities with 518 cases of stillbirth (SB) reported, giving a prevalence of 2.4%. Out 518 women with SB, 331(64%) had macerated stillbirth (MSB) and 187 fresh stillbirth (FSB). The study included all consenting women with stillbirths in the selected health facilities in Mwanza region. Women with a well-established cause like antepartum hemorrhage, hypertensive disorder, labor related, and those with very severe anemia were excluded from the study.

### Sample size estimation and sampling procedures

Sample size was calculated using Kish Lisle formula(1965) 22 using the prevalence of 26% among women with stillbirth due to maternal syphilis in Bolivia 16. A minimum sample size obtained was 295 women. However, 301 women with macerated stillbirth with no established cause were enrolled. Purposive sampling was used to select study sites while convenient sampling was used to select study participants who meet the inclusion criteria until the sample size was reached.

### Recruitment of patients and data collection

Recruitment of participants in this study was done in the gynecology and obstetrics departments. Participants were screened for inclusion criteria and those who met the inclusion criteria were enrolled into the study after informed consent to participate and testing for syphilis was obtained. Relevant information regarding socio-demographic characteristics (age, sex, marital status, educational level, occupation etc.), maternal characteristics (parity, gestation age, antenatal care attendance, pregnancy status i.e. singleton/multiple etc.), foetal characteristics (birth weight) and health system related characteristics (antenatal care (ANC) attendance, ANC booking place) were gathered. All ANC and intrapartum parameters such as level of Haemoglobin, HIV status, syphilis tests and treatment (from antenatal card) and maternal diseases like diabetes mellitus (DM), preeclampsia/eclampsia, hypertension (HTN), mode of delivery, reason for admission, status of the foetus (FSB or MSB), history of obstetric haemorrhage, use of antibiotics, history of previous miscarriage, were collected using a pre-tested structured questionnaire. Preterm birth was defined as any birth before 37 completed weeks of gestation 23.

Under aseptic procedures, about 5 ml of venous blood sample was drawn from each woman into a plain vacutainer tube (Becton, Dickinson and Company, Nairobi, Kenya). Samples were transported to the Microbiology laboratory CUHAS-Bugando whereby blood samples were centrifuged to obtain sera which were stored in cryovials at -80°C until analysis.

### Laboratory procedures

The sera and reagents were brought at room temperature. VDRL testing with 99.7% and 99.6% sensitivity and specificity, respectively was done for all samples as per manufacturer instructions. Those which tested positive were confirmed by *T. pallidum* hemagglutination assay (TPHA) (98.5 sensitivity % and 100% specificity) following manufacturer instructions.

### Statistical data analysis

Statistical data analysis was done using STATA version 13.0. Categorical variables were summarized as proportions and continuous variables were summarized as median with inter-quartile range(IQR). Univariable analysis and multivariable logistic regression models were performed to determine the predictors of *T. pallidum* infections. Variables with p-value less than 0.05 were subjected into multivariable logistic regression analysis and their odds ratios and 95% confidence interval were noted. Variables with p-value of less than 0.05 on multivariable logistic regression analysis were considered as independent predictors.

## Results

### Social Demographic Characteristics

A total of 301 women with macerated stillbirth were enrolled. Of these women, 143(47.5%) and 158 (52.5%) were from rural and urban areas, respectively. The median age of study participants was 27 (IQR: 22 – 34) years. Among these, 164 (54.5%) were multipara. The majority 241 (80.1%) were not married while more than two thirds (71.8%) had primary education level. Twenty-two (7.31%) were HIV seropositive (Table 2).

**Table 2:** Socio-demographic characteristics among 301 women 1 with still birth in Mwanza-Tanzania

*Characteristic*	*Number*	*Percentage*
***Age group***		
15–24	117	38.9
25–34	121	40.2
35–46	63	20.9
***Residence***		
Urban	158	52.5
Rural	143	47.5
***Occupation***		
Employed	26	8.6
Business	45	15.0
Farming	198	65.8
Unemployed	32	10.6
***Education***		
None	32	10.6
Primary	216	71.8
Secondary	38	12.6
Tertiary	15	5.0
***Marital status***		
Married	60	19.9
Not Married	241	80.1
***Gravidity***		
Primigravida	96	31.9
Multigravida	149	49.5
Grandmultgravida	56	18.6
***Parity***		
Primiparous	112	37.2
Multiparous	164	54.5
Grand multiparous	25	8.3
***Gestation Age***		
Preterm	121	40.2
Term	180	59.8
***HIV Status***		
Negative	275	91.5
Positive	22	7.3
Unknown	4	1.3

### Seroprevalence of *T. pallidum* among women with MSB at Mwanza

Among 301 women with stillbirth, 30 (10%, 95% CI: 7.0 – 14.0) were positive for *T. pallidum* using VDRL and after confirmation with TPHA, 18 (6.0%, 95% CI: 3.0–8.0) remained seropositive for *T. pallidum*.

### Factors associated with *T. pallidum* seropositivity among women with stillbirth in Mwanza

On univariable analysis, residing in rural areas (OR: 6.1, 95% CI: 1.7–21.4, p=0.005) and positive HIV serostatus (OR: 4.1, 95% CI: 1.2–13.9, p=0.021) were significantly associated with *T. pallidum* infection. In addition, increase in age (OR: 1.1, 95% CI: 1.0–1.1, p=0.060) and multigravidity (OR: 1.2, 95%CI: 1.0–1.4, p=0.075) were found to have an increased risk of being *T. pallidum* seropositive with a borderline significance. By multivariable logistic regression analysis, residing in rural areas (OR: 5.6, 95% CI: 1.5–20.3, p=0.010) and positive HIV serostatus (OR: 3.9, 95% CI: 1.2–14.1, p=0.036) were found to predict *T. pallidum* seropositivity (Table 3).

**Table 3:** Factor associated with *T. pallidum* infection among women w 1 ith stillbirth in Mwanza

*Patient* *Characteristics*	*T. pallidum*		*Univariate*		*Multivariate*	
	*Positive*	*Negative*	*OR [95% CI]*	*p- value*	*OR [95% CI]*	*p- value*
	*n (%)*	*n (%)*				
***Age***	30 [IQR 24 – 36]	26 [IQR22 – 33]	1.1 [1.0 – 1.1]	0.060	1.0 [0.9 – 1.2]	0.425
***Residence***						
Urban	3 (1.9)	155(98.1)	1.0			
Rural	15 (10.5)	128 (89.5)	6.1 [1.7 – 21.4]	0.005	5.6 [1.5 – 20.3]	**0.010**
***Occupation***						
Employed	2 (7.7)	24 (92.3)	1.0			
Business	0 (0.0)	45 (100.0)	Omitted		-	-
Farming	15(7.6)	183 (92.4)	1.0 [0.2 – 4.6]	0.983	-	-
Unemployed	1 (3.1)	31 (96.9)	0.4 [0.3 – 4.5]	0.449	-	-
***Education***						
None	2 (6.3)	30 (93.8)	1.0			
Primary	14 (6.5)	202 (93.5)	1.0 [0.2 – 4.8]	0.960	-	-
Secondary	1 (2.6)	37 (97.4)	0.4 [0.04 – 4.7]	0.470	-	-
Tertiary	1 (6.7)	14 (93.3)	1.1 [0.09 – 12.8]	0.957	-	-
***Marital status***						
Not Married Married	1 (1.7) 17 (7.1)	59 (98.3) 224 (93.0)	1.0 4.5 [0.6 – 34.3]	0.149	3.6 [0.4 – 29.9]	0.237
***Gravidity***	4 [IQR 1 – 6]	3 [IQR 1 – 5]	1.2 [1.0 – 1.4]	0.075	1.0 [0.5 – 1.9]	0.953
Parity	3 [IQR 1 – 6]	2 [IQR 1 – 4]	1.2 [1.0 – 1.4]	0.073	1.0 [0.8 – 1.3]	0.754
Gestation age	36 [IQR 33 – 38]	37[IQR34 – 38]	1.0 [0.8 – 1.1]	0.566	-	-
***HIV status***						
Negative	14 (5.1)	261 (94.9)	1.0			
Positive	4 (18.2)	18 (81.8)	4.1 [1.2 – 13.9]	0.021	3.9 [1.1 – 14.1]	**0.036**
Unknown	0 (0.0)	4 (100.0)	Omitted			
***Antenatal Visit***						
Yes	17 (5.9)	270 (94.1)	1.0			
No	1 (7.1)	13 (92.9)	0.8 [0.1 – 6.6]	0.851	-	-
***Multiple*** ***Partners***						
No	16 (5.8)	260 (94.2)	1.0			
Yes	2 (8.0)	13 (92.1)	1.4 [0.3 – 6.5]	0.658	-	-

### VDRL test during antenatal period

Out of 301 women with stillbirth, 114 (37.8%) did not test VDRL during antenatal period. Among those who did not test during antenatal period 12 of them (10.5%) were seropositive for *T. pallidum*. Moreover, among those tested negative during antenatal period 3(1.7%) were found to be seropositive. A total of 3 (37.5%) women who tested positive during antenatal remained positive at enrollment (Table 4).

**Table 4:** Status of antenatal VDRL test

VDRL TESTING ANTENATALLY		VDRL Positivity at enrollment
Not tested	114	12(10.5%)
Tested Negative	179	3(1.7%)
Tested Positive	8	3 (37.5%)
**Total**	**301**	**18(6.0%)**

## Discussion

The global burden of stillbirths remains unacceptably high in low- and middle-income countries (LMICs) 3, 24. To the best of our knowledge this is the first study to report seroprevalence of *T. pallidum* among women with MSB in Mwanza, Tanzania. During the study period, a total number of deliveries at all sites were 21,151 and 518(2.4%) were stillborn. Among those, 187 (8.8 per 1000 births) and 331(15.6 per 1000 births) were FSB and MSB, respectively. The overall stillbirth ratio (SBR) per 1000 births in Mwanza region is about 24.5. This rate is lower than values quoted by various studies from the sub-Saharan Africa which reported SBR of 56 and 52.7 per 1000 births 25–27. The lower SBR may reflect improvement in obstetrics care at this setting attributed to the ongoing campaigns of helping baby to breath at one golden minute 14. The rate was much higher compared to 3 to 5 per 1000 births reported from high income countries 3, 5–7, underscoring the importance of improved health care systems in controlling SB.

Seroprevalence of *T. pallidum* in this study was significantly higher than the previous report about five years ago in the same setting (6.0% vs. 2.7%, P <0.04) 17. The possible explanation could be differences in study population whereby the previous study focused on women with normal term pregnancy. This observation shows that *T. pallidum* might play a significant role in adverse pregnancy outcomes including stillbirth in this setting. In addition, it was observed that seroprevalence of *T. pallidum* was significantly higher among women who were not screened during antenatal visits than their counterparts which necessitates the need to put more emphasis on screening these women during antenatal visits. Moreover, 3 women who tested negative during antenatal visits seroconverted giving a seroconversion rate of 1.7% which is slightly lower than seroconversion of 2.7% reported in the previous study in the same settings 17. This emphasizes the need of re-screening these women at delivery or in the third trimester since it takes about 10 – 45 days for *T. pallidum* to be detected in the blood 28, 29 in case of acute infections.

This study further observed that, only VDRL testing was used to diagnose *T. pallidum* infections with no confirmatory test. VDRL is a non-treponemal test hence it detects infection in an indirect way by using cardiolipin antibodies which can result from host cells destructions due to various causes 30–32 showing that VDRL positive results might not necessarily indicative of *T. pallidum* infection. It should be noted that non-treponemal test are sensitive in early detection of *T. pallidum* but it is prone to false positive results which calls for the need to introduce confirmatory test during *T. pallidum* screening in the antenatal settings across the country. Another observation was that some VDRL seropositive women remained seropositive during delivery. This could be explained partial treatment of past infection emphasizing the importance of follow up test after treatment 33.

Among the factors studied, residing in the rural areas was significantly associated with *T. pallidum* seropositivity which is similar to a previous study 29. This could be due to the fact that in rural areas prenatal screening and the availability of the service for screening and treatment is lower compared to urban settings. In this study the majority of women who did not screen for syphilis during antenatal visits were from rural areas this could be due to low socio-economic status and education level, as compared to urban areas.

Another factor which predicted *T. pallidum* seropositivity was positive HIV serostatus which is similar to findings from other reports in Tanzania and Kenya 17, 34, 35. This could be explained by the fact that both HIV and *T. pallidum* are sexually transmitted infection whereby risk factors are also similar. In addition, increased in parity and gravidity were associated with T. pallidum seropositivity, like other previous studies 36, 37. Study done at Ethiopia observed a relatively an age- related downward trend with *T. pallidum* seropositivity with an explanation that could be due to the reflection of the differences in sexual practices, such as number of sexual partners which is common to young women than older women 38.

## Conclusion

The seroprevalence of *T. pallidum* infections is significantly high among women with MSB than in normal pregnant women as previously reported. Residing in rural areas and positive HIV serostatus independently predicted *T. pallidum* seropositivity among women with MSB. A substantial proportion of women with stillbirth seroconverted during the course of pregnancy necessitating the need to introduce re-screening for *T. pallidum* at the beginning of third trimester (28 weeks) or at delivery. Confirmatory test (Treponemal test) should be considered during antenatal screening of *T. pallidum* infection to avoid unnecessary false positive results and unnecessary treatment.

## List of Abbreviation

ANC: Antenatal Clinic
BMC: Bugando Medical Centre
CI: Confidence Interval
CREC: CUHAS/BMC Research Ethics and Review Committee
CUHAS: Catholic University of Health and Allied Sciences
DM: Diabetes mellitus
FSB: Fresh Still Birth
HIV: Human Immunodeficiency Virus
HTN: Hypertension
IQR: Interquartile range
LMICs: Low and Middle Income Countries
MSB: Macerated Stillbirth
SDDH: Sengerema Designated District Hospital
TPHA: Treponema pallidum hemagglutination test
OR: Odds Ratio
VDRL: Venereal Disease Research laboratory

## Declaration

### Availability of data and material

All data generated/ analysed during this study are included in this manuscript.
